# Supplementary material for: Human PARP1 Facilitates Transcription through a Nucleosome and Histone Displacement by Pol II In Vitro
Source: Int J Mol Sci. 2022 Jun 26;23(13):7107. doi: 10.3390/ijms23137107 (PMC9266421; doi:10.3390/ijms23137107)
Supplement: Supplementary file 1 [file ijms-23-07107-s001.zip › ijms-1733323-supplementary.pdf]

Human PARP1 Facilitates Transcription through a Nucleosome and Histone Displacement by Pol II *in vitro*

Elena Kotova, Fu-Kai Hsieh, Han-Wen Chang, Natalia V. Maluchenko, Marie-France Langelier, John M. Pascal, Donal S. Luse, Alexey V. Feofanov and Vasily M. Studitsky

SUPPLEMENTARY FIGURES

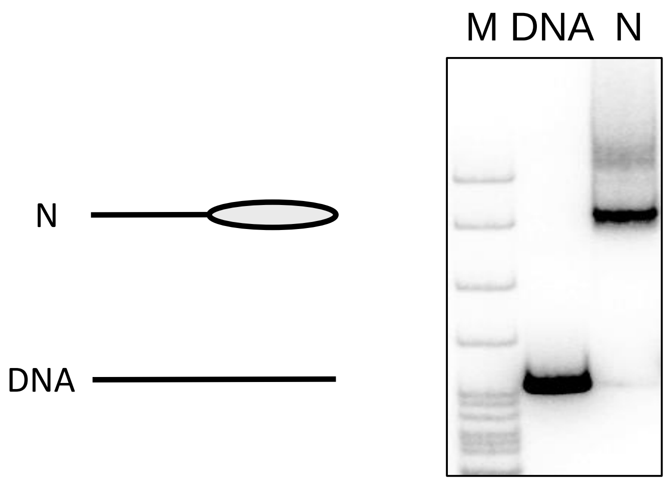

Figure S1. Characterization of nucleosomes for transcription by Pol II. Nucleosomes were reconstituted on DNA-end-labeled 603 templates and analyzed by 4% native PAGE. Positions of mononucleosomes (N) and histone-free DNA in the gel are indicated. M – end-labeled pBR322-MspI digest.

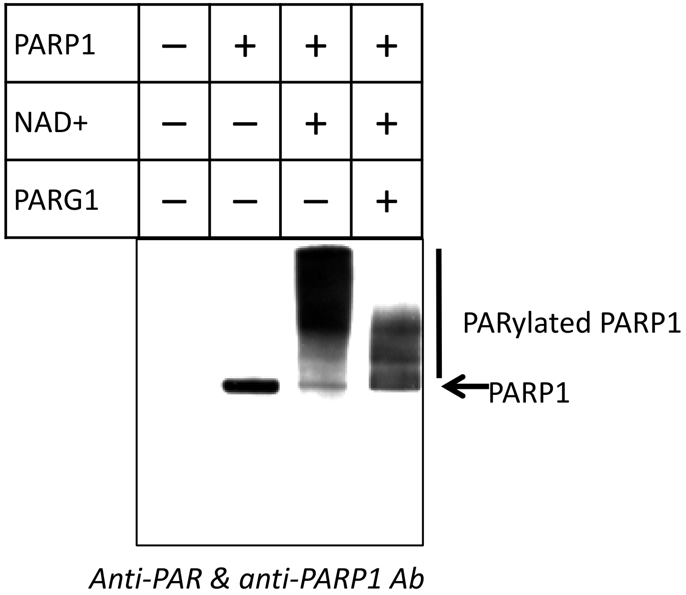

Figure S2. PARP-1 is automodified in the presence of NAD+. The transcription reactions were conducted as described in Figure 1a in the presence or absence of PARP1, NAD and/or PARG. The proteins were separated by an SDS-PAGE and transferred to a membrane. The Western blot was stained with anti-PARP1 antibodies and antibodies to poly(ADP)-ribose (PAR). Positions of PARP1 and PARylated PARP1 are indicated.

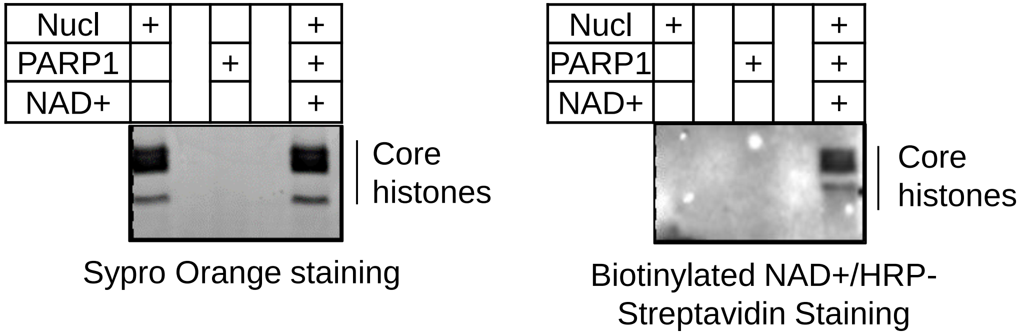

Figure S3. Core histones are modified in the presence of PARP1 and bio-NAD+. Nucleosomes were incubated in the transcription buffer in the presence or absence of PARP1 and/or bio-NAD+. The proteins were separated by an SDS-PAGE, stained with Sypro Orange and transferred to a membrane. The membrane was stained with horse radish peroxidase (HRP)-conjugated streptavidin. Positions of core histones are indicated.

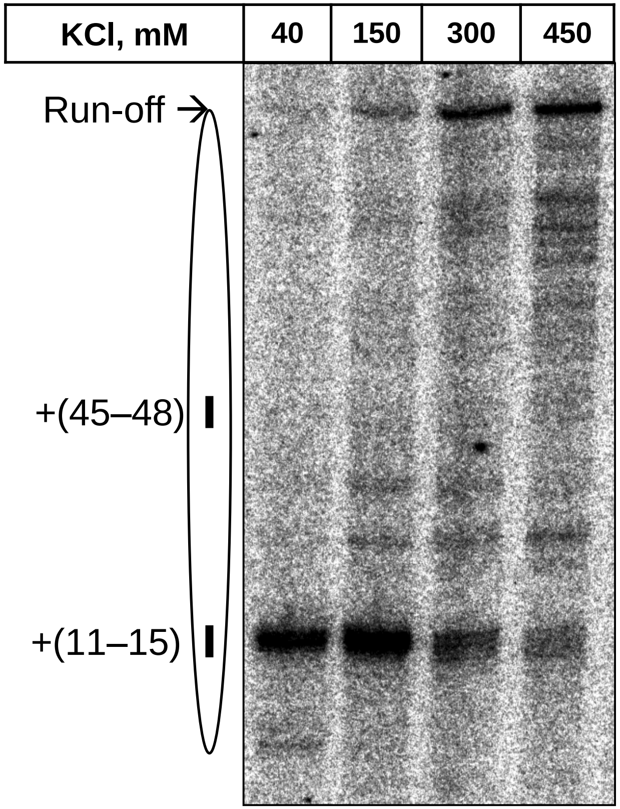

Figure S4. The 603 nucleosomes form a high barrier to human Pol II. hPol II elongation complexes were assembled and the nucleosomes transcribed in the presence of various concentrations of KCl as described in Figure 1a. Analysis of pulse-labeled RNA by denaturing PAGE. The positions of the paused and the run-off transcripts in the gel are indicated.

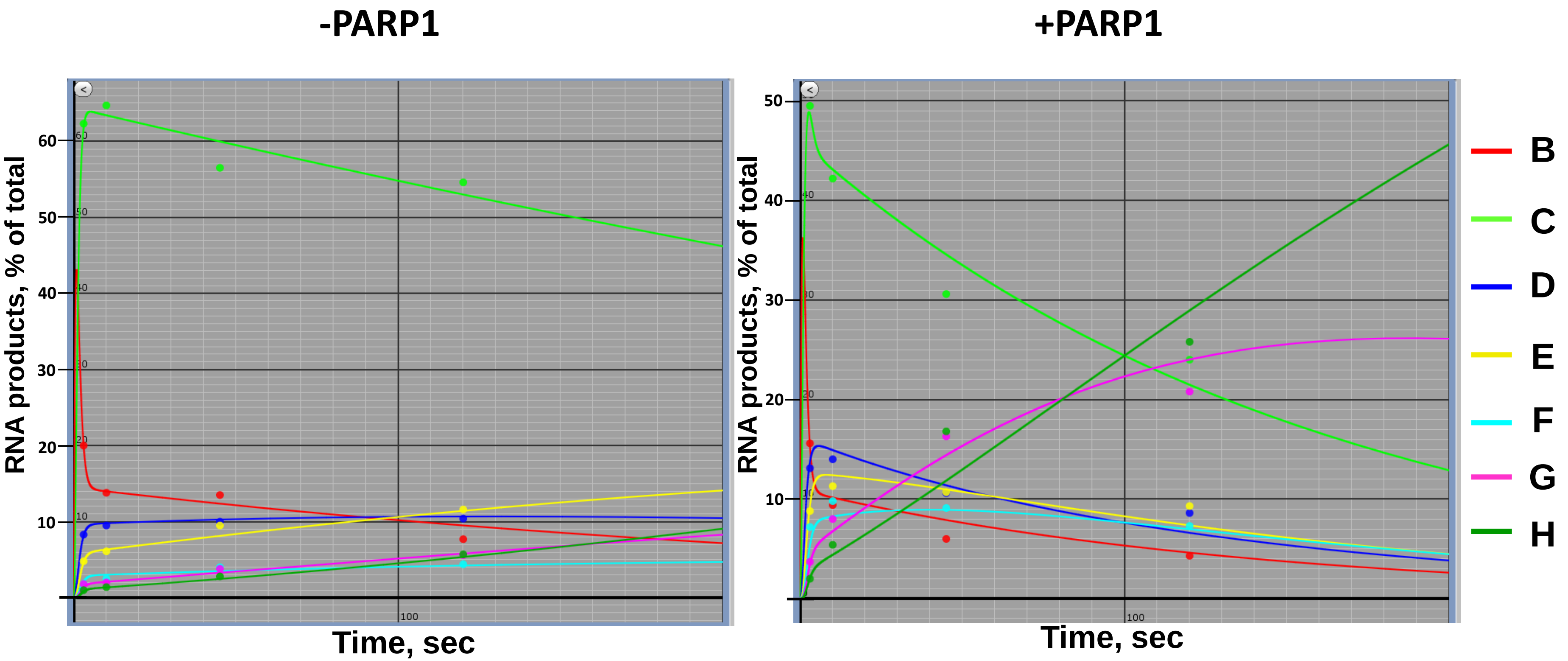

Figure S5. Analysis of time-courses of transcription through chromatin in the presence or in the absence of hPARP using KinTek software: fit with the model.

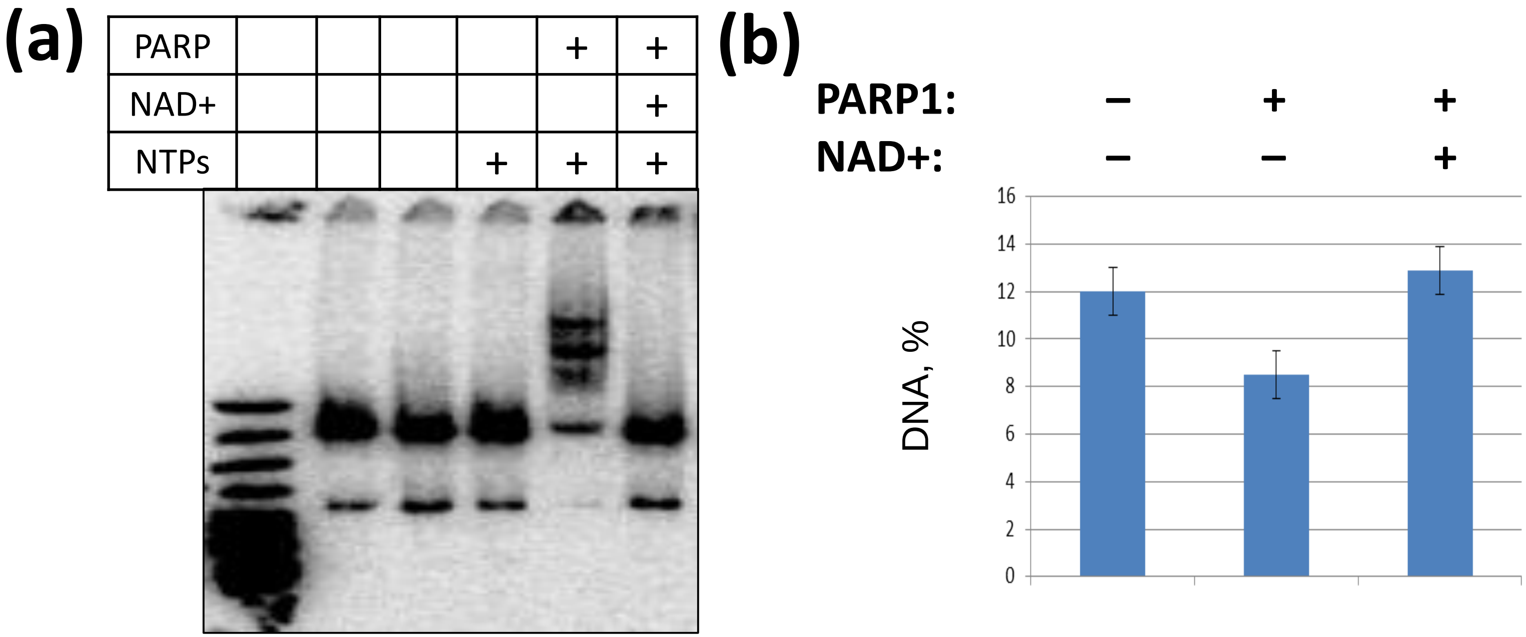

Figure S6. PARP1 does not induce DNA displacement from nucleosomes in absence of transcription. **(a)** Nucleosomes assembled on end-labeled DNA were incubated in the presence of PARP1, NTPs and/or NAD+ in the absence of Pol II as described in Figure 4a. Analysis in non-denaturing gel. **(b)** The amounts of histone-free DNA produced after transcription was quantified as described in Figure 4b.
